# Variation of Soil Aggregation along the Weathering Gradient: Comparison of Grain Size Distribution under Different Disruptive Forces

**DOI:** 10.1371/journal.pone.0160960

**Published:** 2016-08-16

**Authors:** Yujie Wei, Xinliang Wu, Jinwen Xia, Xue Shen, Chongfa Cai

**Affiliations:** 1 Key Laboratory of Arable Land Conservation (Middle and Lower Reaches of Yangtze River) of the Ministry of Agriculture, Soil and Water Conservation Research Centre, College of Resources and Environment, Huazhong Agricultural University, Wuhan, China; 2 College of Economics and Management, Huazhong Agricultural University, Wuhan, China; University of Vigo, SPAIN

## Abstract

The formation and stabilization of soil aggregates play a key role in soil functions. To date, few studies have been performed on the variation of soil aggregation with increasing soil weathering degree. Here, soil aggregation and its influencing factors along the weathering gradient were investigated. Six typical zonal soils (derived from similar parent materials) were sampled from temperate to tropical regions. Grain size distribution (GSD) in aggregate fragmentation with increasing disruptive forces (air-dried, water dispersion and chemical dispersion) was determined by laser diffraction particle size analyzer. Different forms of sesquioxides were determined by selective chemical extraction and their contributions to soil aggregation were identified by multiple stepwise regression analysis. The high variability of sesquioxides in different forms appeared with increasing free oxide content (Fe_d_ and Al_d_) from the temperate to tropical soils. The transformation of GSD peak to small size varied with increasing disruptive forces (p<0.05). Although in different weathering degrees, zonal soils showed a similar fragmentation process. Aggregate water stability generally increased with increasing soil weathering (p<0.01), with higher stability in eluvium (A) horizon than in illuvium (B) horizon (p<0.01). Crystalline oxides and amorphous iron oxides (Fe_o_), especially (Fe_d_-Fe_o_) contributed to the formation of air-dried macroaggregates and their stability against slaking (R^2^ = 55%, p<0.01), while fine particles (<50μm) and Fe_o_ (excluding the complex form Fe_p_) played a positive role in the formation of water stable aggregates (R^2^ = 93%, p<0.01). Additionally, water stable aggregates (including stability, size distribution and specific surface area) were closely related with pH, organic matter, cation exchange capacity (CEC), bulk density (BD), and free oxides (including various forms) (p<0.05). The overall results indicate that soil aggregation conforms to aggregate hierarchy theory to some extent along the weathering gradient and different forms of sesquioxides perform their specific roles in the formation and stabilization of different size aggregates.

## Introduction

Soil aggregation plays a vital role in soil functions, such as infiltration capacity, tilth, gas exchange, organic matter stabilization and erodibility [1, 2]. Soil aggregation is the result of the interactions of numerous physical, chemical and biological factors with intricate feedback mechanisms [3–5]. Understanding the mechanism of soil aggregation is key to improve soil quality and facilitates to the management of soil biogeochemical processes.

Soil organic matter (SOM) and sesquioxides are widely accepted as the main organic and inorganic agents influencing soil aggregation [4, 6–8]. Duiker et al. demonstrated that poorly crystalline iron oxides are more effective than free forms in stabilizing soil aggregates [9]. Mbagwu and Schwertman suggested that aluminum oxides were more effective in aggregation of gibbsite than iron oxides [10]. Barthès et al. reported that Al-containing sesquioxides have a more important role than soil organic matter in the aggregation of tropical soils [7]. Peng et al. found that Fe/Al oxides were likely the major agents in micro-aggregates while SOM plays a primary role in stabilizing the macro-aggregates in Ultisols [11]. From the aforementioned reports, it can be seen that these agents vary in their functions in aggregation. As numerous factors are involved soil aggregation, it is usually difficult to distinguish their specific role separately [11, 12]. For a better investigation of the specific effects of sesquioxides and organic matter on soil aggregation, other factors should be kept as constant as possible.

In the past decades, several mechanisms of aggregation have been proposed [4, 13–15]. According to the widely-accepted hierarchical theory of aggregation, aggregates are formed in different stages with a different bonding mechanism for each stage, and micro-aggregates join together to form macroaggregates [14]. Aggregate hierarchy has been well studied in relation to organic matter, clay content and types in many temperate soils (such as Luvisols, Mollisol), but not in the highly weathered soils with rich sesquioxides (tropical and subtropical soils) [3, 5, 7, 11].

The fundamental criterion to identify soil aggregation is the stepwise breakdown of aggregates with increasing dispersion [3, 16]. Water and Oades stressed that aggregate hierarchy covers a range of aggregates of different sizes [3], implying that it is necessary to obtain the detailed information about the aggregate or particle size distribution from nanometer-micrometer to millimeter in soil aggregation analysis. However, the traditional sieve-pipette method used in previous studies is not appropriate for assessing the continuous aggregate size distribution with a wide range of scales, due to the limited information on grain size distribution [17], which hinders the examination of submicron-scale aggregate structure and the possible presence of hierarchy at a small scale [5]. In recent years, laser diffraction (LD) technique has been proved to be a powerful method for the analysis of grain size distribution in a wide range of scales, particularly in data detection at clay-size scale [18–20]. Currently, to our knowledge, LD method is mainly applied to test soil particle size distribution or microaggregate in a narrow range, and rarely used in aggregate size distribution from submicron to millimeter.

Highly weathered soils are important farmland resources, and are widely distributed in the subtropical-tropical areas. These soils are usually characterized by different weathering degrees and different forms of sesquioxides due to the variation of climate condition. Generally, soils from the temperate and tropical regions displayed an increasing soil weathering degree [21], but little information is available about the aggregation of these soils along the weathering gradient, and their relationships with sesquioxides and other properties. In the present study, six typical zonal soils with various weathering degrees were selected as research objects. Gain size distributions (0.1~3000μm) under increasing disruptive forces (air-dried, water dispersion, chemical dispersion) were systematically analyzed by laser diffraction particle size analyzer, and different forms of active sesquioxides were determined by chemical selective extraction techniques. The main objectives of this study were: (i) to compare grain size distributions under different treatments and further check the soil aggregation process; (ii) to identify the major factors that affect soil aggregation along the weathering gradient. These results will supplement the theoretic aggregation mechanism of soils in subtropical-tropical regions. All the abbreviations used in this paper are summarized in Table 1.

**Table 1:** Summary of abbreviations in this article.

Abbreviation	Full name
Fe_d_, Al_d_	Free iron and aluminum oxides
AI	Aggregation index
AD	Air dried treatment
BD	Bulk density
CD	Chemical dispersion treatment
CEC	Cation exchange capacity
C_u_	Uniformity coefficient
C_c_	Curvature coefficient
CV	Coefficient of variation
DI	Detachability index
Fe_o_, Al_o_	Amorphous iron and aluminum oxides
Fe_p_, Al_p_	Complex iron and aluminum oxides
Fe_d_-Fe_o_, Al_d_-Al_o_	Crystalline iron and aluminum oxides
Fe_o_-Fe_p_, Al_o_-Al_p_	Amorphous iron and aluminum oxides excluding the complex form
GSD	Grain size distribution
LD	Laser diffraction technique
MVD	Mean volume diameter
PSD	Particle size distribution
SOM	Soil organic matter
SSA	Specific surface area
VIF	Variance inflation factor
WD	Water dispersion treatment

## Materials and Methods

We declare that all soil samplings were performed under the permission of the owners of the farmlands and the field studies did not involve endangered or protected species.

### Study site and soil sampling

**Fig 1:**
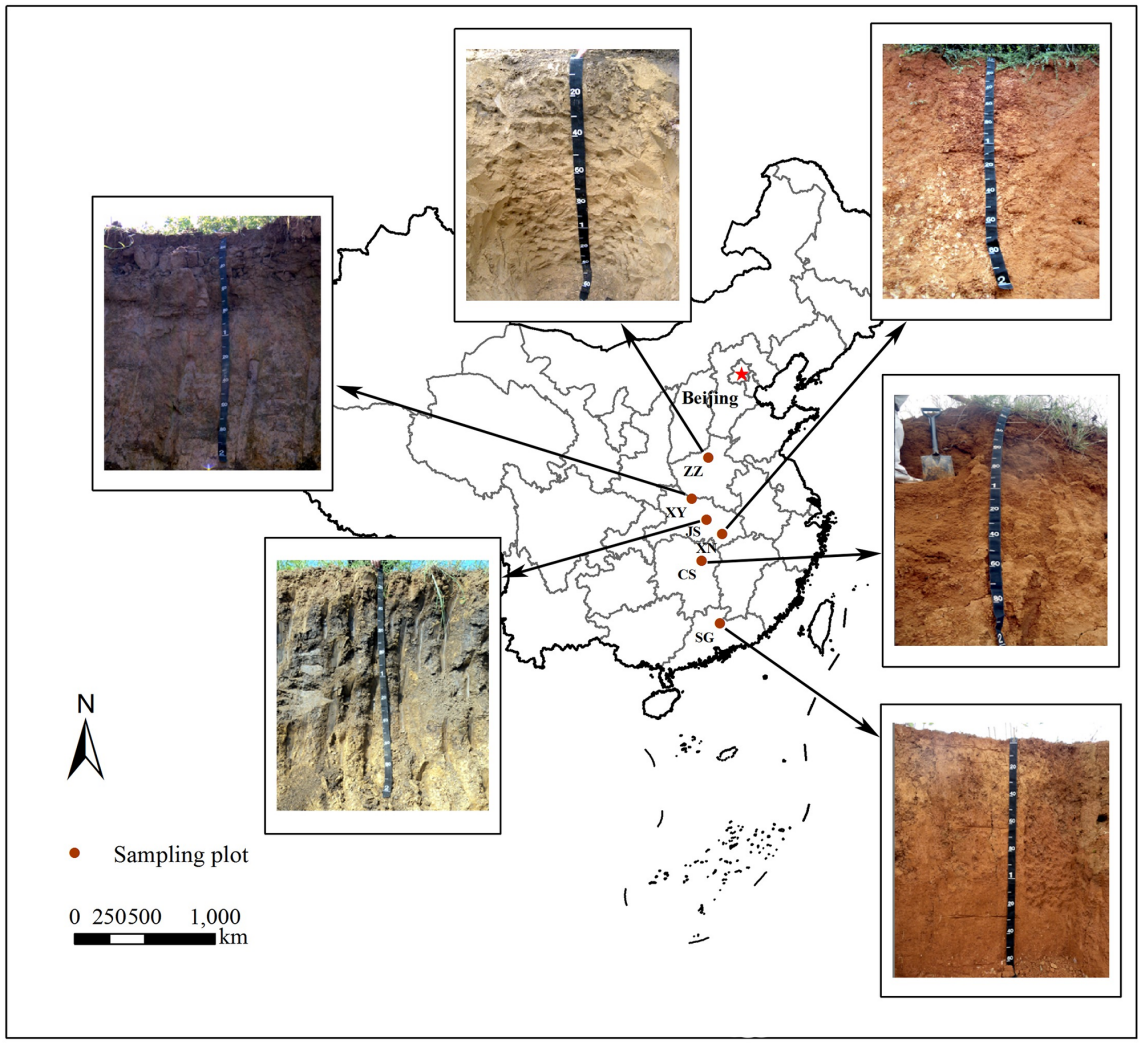
Location and soil profiles of selected typical zonal soils in central-south China.

For a better understanding of soil aggregation, five typical zonal soils derived from quaternary clay and one temperate soil were selected in a similar soil texture. These soils from central and south China covered three monsoon climatic regions (temperate, subtropical and tropical monsoon climate). The mean annual temperature and precipitation ranged from 14 to 20°C and from 640 to 1778 mm respectively, indicating an increasing trend of water and heat conditions contributing to soil weathering. The sampling sites were separately located in Zhengzhou City (ZZ) in Henan province, Xiangyang City (XN), Jingshan County (JS), Xianning City (XN) in Hubei province, Changsha City (CS) in Hunan province, Shaoguan City (SG) in Guangdong province, China. The soils ZZ derived from alluvium were classified into Cambosols, XY, JS and XN into Argosols, CS into Ferrosols, and SG into Ferralosols according to Chinese Soil Taxonomy [22], which were Cambisols (ZZ), Luvisols (XY and JS), Alisols (XN and CS) and Acrisols (SG) according to the WRB (2014). All sampling sites were located on gentle slopes or plain with the slope gradient smaller than 5%, and were cultivated by human beings with intact genetic soil profile and slight erosion. Detailed information of soil sampling sites including locations, climates, farming systems, topographies, soil groups and soil profiles is listed in Table 2 and Fig 1 (S1 Fig).

**Table 2:** Information of the studied zonal soils.

Code	Longitude /latitude	MAT/°C	MAP/mm	Topography	Land use	Clay mineralogy	Soil taxonomy
ZZ	113°32′E/ 34°54′N	14.2	641	Plain	Maize/wheat	Ill- Kao-Ver	Ochri-Aquic Cambosols
XY	112°09′E/ 32°19′N	16.0	878	Gentle slope	Maize	Ill- Kao-Ver	Ferri-Udic Argosols
JS	113°14′E/ 30°57′N	16.3	1179	Gentle slope	Soybean	Ill-Kao-1.4nm	Ferri-Udic Argosols
XN	114°22′E/ 30°00′N	16.8	1577	Gentle slope	Tea plantations	Kao- Ill	Ferri-Udic Argosols
CS	112°46′E/ 28°22′N	17.0	1422	Gentle slope	Waste land	Ill-Kao-1.4nm	Argi-Udic Ferrosols
SG	113°56′E/ 24°18′N	20.4	1778	Gentle slope	Potato	Kao- Ill	Hi-weatheri-Udic Ferralosols

MAT, mean annual temperature; MAP, mean annual precipitation. Kao = kaolinite; Ill = illite; Ver = vermiculite; 1.4nm = 1.4nm intergrade mineral. Soil taxonomy determined according to Chinese Soil Taxonomy (2001).

At each site, a soil profile pit was dug, and soil samples were taken from two distinct profile horizons, namely eluvium (A) and illuvium (B), from July to September in 2012. Undisturbed soil cores (100 cm^3^) were taken for the analysis of bulk density. As previously reported, the structure of aggregates smaller than 3 mm was not influenced by cropping history or other external forces, but by the nature and proportions of soil constituents [23]. Therefore, in this study we used soils smaller than 2 mm as experimental objects. After collection, the field-moist disturbed soil samples were gently broken up manually so that the large clods broke along natural fissures, and then fully air-dried and ground through 2 mm sieve after the removal of large roots and other fresh organic materials.

### Soil analyses

Soil basic properties were determined using standard analytical methods: bulk density (BD) by the core method; soil pH in a 1:2.5 soil/water mass ratio by the pH meter; soil organic matter (SOM) by acid potassium-dichromate (K_2_Cr_2_O_7_, *c* = 0.4M) digestion; cation exchange capacity (CEC) by ammonium acetate exchange method. [24] Free Fe and Al oxides (Fe_d_ and Al_d_) were extracted by dithionite-citrate-bicarbonate (DCB) [25]; amorphous Fe and Al oxides (Fe_o_ and Al_o_) by acid ammonium oxalate [26]; complex Fe and Al oxides (Fe_p_ and Al_p_) by sodium pyrophosphate [27]. The Fe and Al extracted by the above-mentioned procedures were determined by ICP-OES (VISTA-MPX, Varian, America) after dilution. All analyses were run in triplicate and averaged for statistical analysis (Table 3). In addition, we calculated the contents of crystalline oxides (Fe_d_-Fe_o_ and Al_d_-Al_o_) and the amorphous oxides excluding complex oxides (Fe_o_-Fe_p_ and Al_o_-Al_p_) by the difference between free and amorphous oxides, and between amorphous and complex oxides, respectively [28]. These indices would be selected for further multiple stepwise regression analysis.

**Table 3:** Basic physicochemical properties of studied soils.

Code	Horizon/depth(cm)	pH	SOM (g kg^-1^)	CEC(cmol kg^-1^)	BD(Mg m^-3^)	Fe_d_(g kg^-1^)	Al_d_(g kg^-1^)	Fe_o_(g kg^-1^)	Al_o_(g kg^-1^)	Fe_p_(g kg^-1^)	Al_p_(g kg^-1^)
ZZ	A/0-20	7.47	16.88	9.87	1.39	7.63	1.13	1.78	1.09	0.07	0.08
	B/20-	7.93	3.17	6.20	1.38	7.02	1.04	0.74	0.90	0.03	0.05
XY	A/0-40	6.86	9.70	20.65	1.65	17.70	3.22	2.60	2.51	0.05	0.20
	B/40-	7.38	5.34	21.52	1.61	19.62	3.71	1.73	2.23	0.03	0.18.
J S	A/0-20	7.09	18.42	20.29	1.43	27.37	5.20	4.53	2.31	0.18	0.36
	B/20-	7.31	10.48	22.85	1.61	29.83	5.22	2.16	2.36	0.04	0.21
XN	A/0-20	4.62	20.67	10.35	1.47	25.56	5.08	2.79	2.51	0.35	1.04
	B/20-	5.79	6.99	12.03	1.36	56.54	6.14	2.59	3.14	0.08	1.12
CS	A/0-30	6.13	16.66	15.85	1.46	40.94	9.56	4.17	3.54	0.07	0.99
	B/30-	6.45	5.12	16.45	1.39	40.83	11.88	3.75	3.43	0.07	0.81
SG	A/0-30	5.71	22.43	9.97	1.25	87.15	14.94	3.96	2.43	0.31	0.47
	B/40-	6.12	12.23	11.26	1.24	150.35	21.21	4.34	2.24	0.08	0.35
	Mean	6.57	12.34	14.77	1.44	42.54	7.36	2.93	2.39	0.11	0.49
	CV	14%	53%	38%	9%	95%	82%	41%	33%	96%	81%

Abbreviations: SOM, soil organic matter; CEC, cation exchange capacity; BD, bulk density; Fe_d_ and Al_d_, free Fe and Al oxides; Fe_o_ and Al_o_, amorphous oxides; Fe_p_ and Al_p_, complex Fe and Al oxides; CV, coefficient of variation.

### Laser diffraction analyses

Selected soils (<2mm) were pretreated using the following three treatments: (i) air-dried (AD); (ii) water dispersion (WD), 1g of each samples was immersed in water for 24 h and then shaken for 2 h; (iii) chemical dispersion (CD), 0.3~0.5g of each sample was fully-dispersed in dispersant (0.5mol L^-1^sodium hydroxide or sodium oxalate). Following each treatment, grain (including aggregates and particles) size distribution was analyzed using the Mastersizer3000 equipment, which measures the volume content (%) in 100 bin distribution ranging from 0.01 to 3000μm. In the three treatments, AD sample was measured with laser diffraction technique in dry mode (the particle absorption index was 0.3, particle refractive index was 1.60 and dispersant refractive index was 1) and the other two treatments (WD and CD) in wet mode (the particle absorption index was 0.1, particle refractive index was 1.60 and dispersant refractive index was 1.33) [19, 29]. A background measurement was performed firstly to subtract the ambient light signal from the total scattered light received from the sample, followed by five consecutive analyses of soil sample per lens. The data of five measurements were averaged to obtain relative volume data. In this study, it is assumed that the volume contents of grain sizes measured by the dry and wet mode in laser diffraction technique are comparable to the previous studies [19, 20]. Besides, we also obtained other parameters, such as specific surface area (SSA) and characteristic grain size (d_10_, d_30_ and d_60_) [30]. d_10_, d_30_ and d_60_ denote constrained grain size, median grain size and effective grain size respectively, which referred to the diameters corresponding to the soil cumulative volumes of 10, 30 and 60%. They could be used to describe soil grain size distributions.

The indices, uniformity coefficient (C_u_) and curvature coefficient (C_c_), were calculated based on these three characteristic grain sizes to describe the uniformity of soil grain size distribution [30]:

$$C_{\text{u}}=\frac{d_{60}}{d_{10}}$$ (1)

$$C_{\text{c}}=\frac{d_{30}^{2}}{d_{10}\times d_{60}}$$ (2)

The larger C_u_ value indicates the broader range of GSDs and higher unevenness of grains. C_c_ reflects the entire morphology of grain size cumulative curve, especially the distribution of the grains ranging from d_10_ to d_60_.

Mean volume diameter (MVD, mm) was calculated as follows:

$$\text{MVD}=\sum_{1}^{100}r_{i}\times v_{i}$$ (3)

Where *v*_*i*_ (%) is the volume percentage of the grains in the bin *i* with the average diameter *r*_*i*_ (mm). MVD_AD_, MVD_WD_ and MVD_CD_ denote the mean volume diameters in AD, WD and CD treatments. To evaluate the fragmentation degree of air-dried aggregates after being immersed in water and the aggregation degree of water stable aggregates from particles, detachability index (DI) and aggregation index (AI) were calculated by the following formulas [31]:

$$\text{DI}=\frac{{\text{MVD}}_{\text{AD}}-{\text{MVD}}_{\text{WD}}}{{\text{MVD}}_{\text{AD}}}\times 100$$ (4)

$$\text{AI}=\frac{{\text{MVD}}_{\text{WD}}-{\text{MVD}}_{\text{CD}}}{{\text{MVD}}_{\text{WD}}}\times 100$$ (5)

### Data analysis

To investigate the similarity of soil aggregation among these zonal soils in different weathering degrees, cluster analysis was conducted on the volume differences (ΔV, %) of different size grains between the treatments of WD and AD, and between CD and WD, respectively. The Euclidean square distance was adopted and the soil samples were classified into three clusters.

The differences among the selected soils were statistically analyzed by three-way ANOVA (p<0.05). Normality tests were carried out for all variables using the Shapiro-Wilk method. The variables not conforming to normal distribution were transformed by natural logarithmic treatment. Pearson’s correlations were made among variables at the level of p<0.05, 0.01 and 0.001. Multiple stepwise regressions were performed between soil aggregate stability (MVD_WD_, SSA_WD_, C_u_, C_c_, DI and AI) and basic properties (pH, BD, SOM, CEC, SSA_CD_, >50μm, 2~50μm, <2μm, MVD_CD_, different forms of sesquioxides including Fe_d_-Fe_o_ and Al_d_-Al_o_, Fe_o_-Fe_p_ and Al_o_-Al_p_, Fe_p_ and Al_p_) to determine the variables accounting for the majority of soil aggregation indices. Besides, the variance inflation factor (VIF) was adopted to evaluate the collinearity between the explanatory variables. The VIF <5 indicated the weak collinearity. All data analyses were performed using the SPSS16.0 [32].

## Results

### Physicochemical properties

The basic physicochemical properties of the studied soils are listed in Table 3. The selected soils generally exhibited alkaline to strongly acidic from the central to south China, and the pH of B horizon was higher than that of A horizon. Soil organic matter content was lower in B horizon than in A horizon, and the same trend was observed in CEC except for ZZ. Bulk density showed the least variation with CV of 9% among all the tested properties. The ranges of Fe_d_ and Al_d_ were 7.02~150.35g kg^-1^and 1.04~21.21 g kg^-1^ separately with the CVs of 95% and 82%. The contents of free oxides increased significantly from central to south China and Fe_d_ was remarkably higher than Al_d_, indicating the increase of the soil weathering degree from ZZ to SG [21]. Amorphous oxides (Fe_o_ and Al_o_) varied in a narrow range with the CVs of 41% and 33%. Complex oxides also exhibited high variations, especially Fe_p_ with a CV as high as 96%; besides, the content of Fe_p_ was much lower than that of Al_p_.

### Grain size distribution

**Fig 2:**
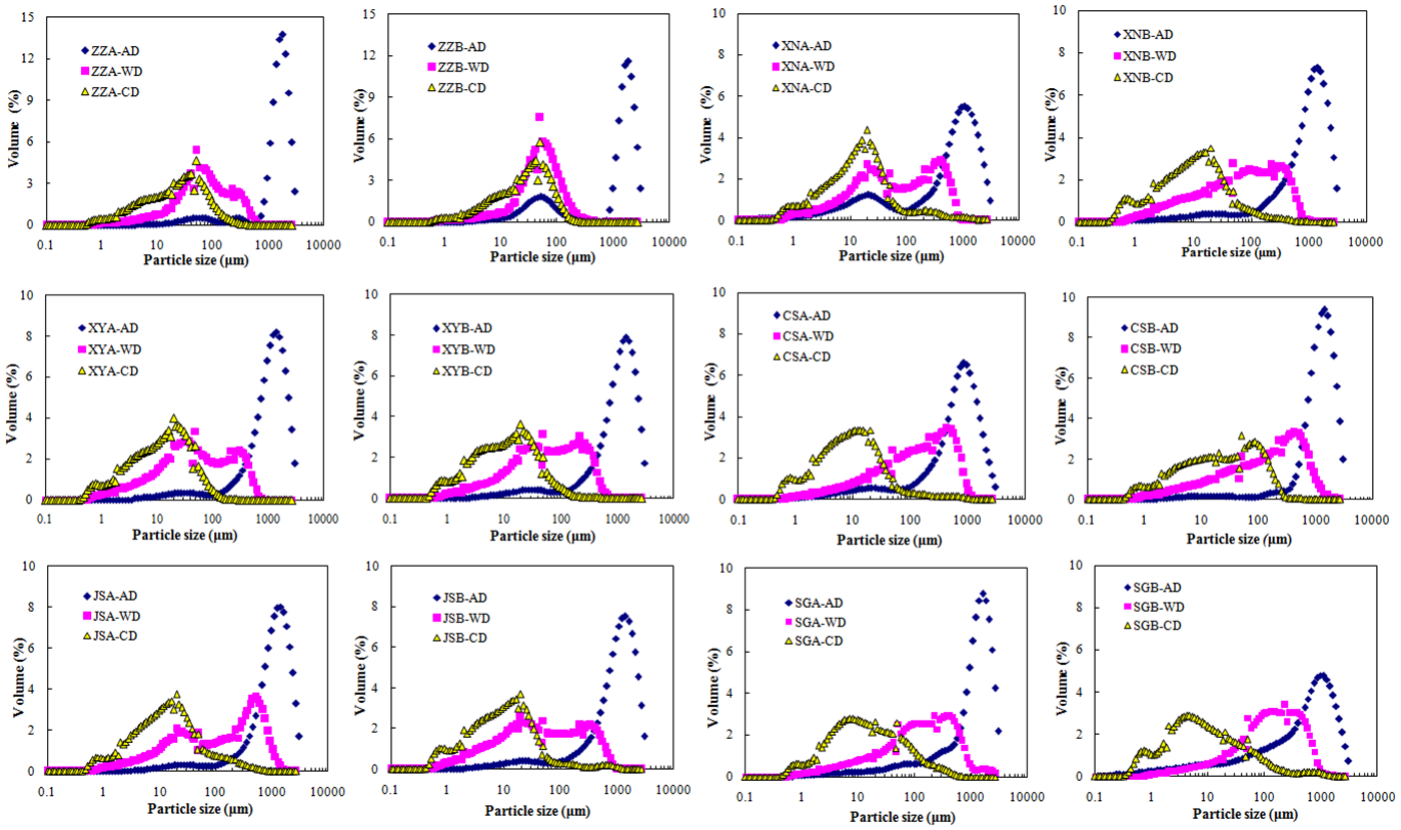
Grain size distributions of zonal soils selected under different treatments. Blue diamond, air-dried soils (AD); red-square, water dispersion (WD); yellow-triangle, chemical dispersion (CD).

Grain-size distributions (GSDs) of the selected soils under three treatments are shown in Fig 2. Air-dried aggregates were characterized by large size (>250 μm) and the main peak volume contents were observed in the range from 860 to 1850 μm. For ZZ-B and XN-A, small secondary peaks were observed at 50 μm and 20 μm. The existence of aggregates larger than 2000 μm in all soils suggested that not all dry aggregates were rigidly spherical, but prismatic or columnar with the long axis exceeding 2000 μm. In all these soils, C_u_ and C_c_ of ZZ-A, XY-A, XY-B, JS-A, JS-B, CS-B were below 10 and 3 respectively, reflecting narrow distributions of the macroaggregates, and the higher content of microaggregates in others soils (Table 4).

**Table 4:** Parameters of grain distributions under different treatments (AD, WD and CD).

Code	AD		WD		CD	
	C_u_	C_c_	C_u_	C_c_	C_u_	C_c_
ZZA	4.6	2.2	6.3	1.3	10.8	1.0
ZZB	47.6	17.1	4.4	1.4	8.4	1.3
XYA	4.7	1.6	11.5	1.2	9.2	1.0
XYB	6.9	2.1	12.6	1.0	9.0	0.8
JSA	5.1	1.6	18.8	0.7	7.6	1.1
JSB	8.0	2.1	15.3	0.9	8.7	0.9
XNA	73.9	6.5	14.1	0.8	8.7	1.3
XNB	11.1	2.7	16.6	1.1	10.7	1.2
CSA	19.5	4.6	13.6	1.2	7.9	1.0
CSB	3.1	1.1	23.8	1.1	11.3	0.9
SGA	14.8	4.0	19.4	1.7	8.7	0.8
SGB	65.7	3.6	12.3	1.5	10.1	0.9

Abbreviations: AD, air dried; WD, water dispersion; CD, chemical dispersion; C_u_, uniformity coefficient; C_c_, curvature coefficient.

**Fig 3:**
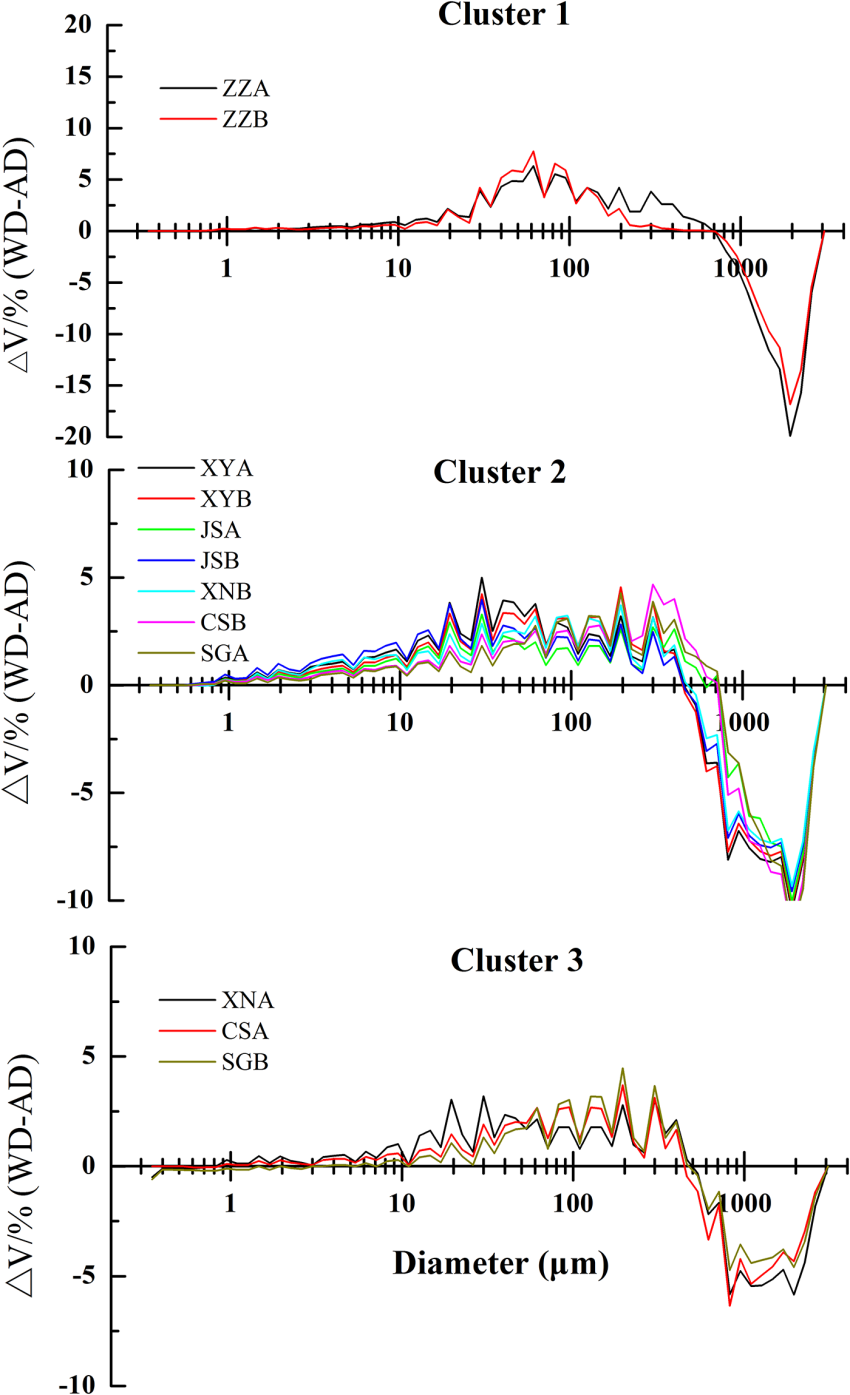
Differences in volume (Δ*V*, %) between the water dispersion (WD) and air-dried (AD) treatments for the zonal soils in three clusters.

When subjected to slaking in water, the studied soils displayed significant aggregate disruption (Fig 3). The peaks of GSDs in WD treatment shifted to smaller sizes to some extent relative to AD treatment. Soils XY, XN and JS-B mostly exhibited distributions of double peaks and most of water-stable aggregates were mainly concentrated in the range of 20~600 μm. Except that in ZZ-A and ZZ-B, the C_u_ above 10 in the other soils indicated the broad range and unevenness of water-stable aggregate distribution (Table 4).

Large amounts of macroaggregates (>700 μm) broke down into smaller aggregates and particles (>10 μm), with the temperate ZZ soils being around 60 μm, and less macroaggregates (>500 μm) of XN-A, CS-A and SG-B were fragmented into less aggregates or particles of a similar size (>10 μm) (Fig 3). The difference of the aggregate fragmentation between the second cluster and the other two clusters was in the amount of broken macroaggregates and the homogeneous distribution of the yielded materials (>2 μm).

**Fig 4:**
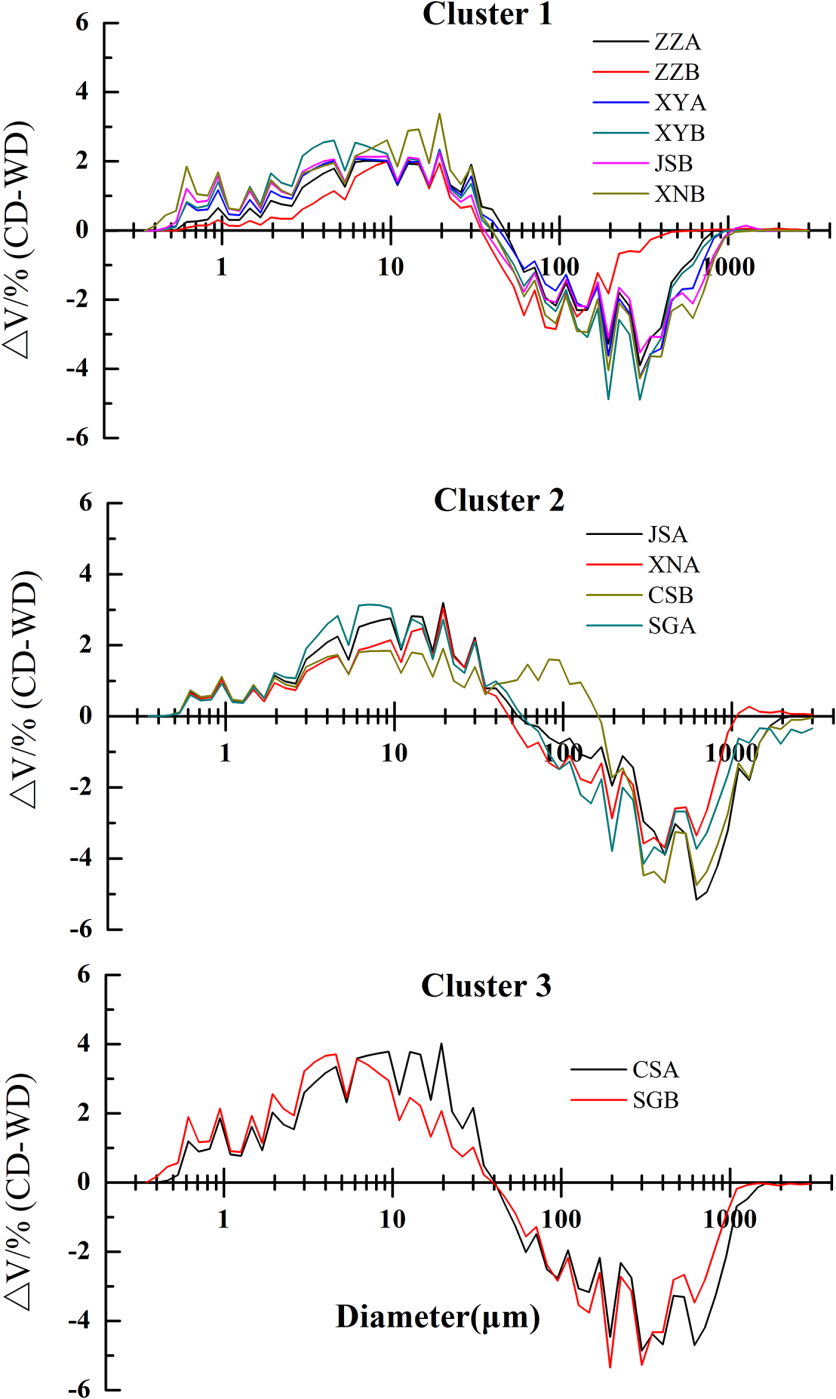
Differences in volume (Δ*V*, %) between the chemical dispersion (CD) and water dispersion (WD) treatments for the zonal soils in three clusters.

Compared with WD treatment, all the curves of particle size distributions (PSDs) in CD treatments shifted to smaller sizes in a certain degree (Fig 2). The peaks of PSDs for ZZ soils were situated at 40 μm, and those for other soils were located in the range of 5 to 20 μm. C_u_ (7.6~11.3) and C_c_ (0.8~1.3) of PSDs indicate a similar narrow range of particle sizes among these zonal soils. Based on the similarity in the fragmentation process, these zonal soils were grouped into three clusters as well (Fig 4). In the first cluster, the water stable aggregates of XY-A, XY-B, JS-B and XN-B showed a similar fragmentation process to that of ZZ-A and ZZ-B, which indirectly reflects their similarity in aggregations of primary particles to form water stable aggregates although in different weathering degree. In the second cluster, there were more large size water stable aggregates (>50 μm) (JS-A, XN-A, CS-B and SG-A) than in the first cluster, suggesting more fine particles (0.4~50 μm) were correspondingly released in the former cluster, and this phenomenon was more remarkable for soils in the third cluster.

### Soil aggregate stability

For air-dried soils, mean volume diameters (MVDs) varied from 0.72 to 1.61 mm with a CV of 22% and the soil surface specific area (SSA) ranged from 9 to 164 m^2^ kg^-1^. The data of MVD_AD_ and SSA_AD_ provided the initial reference in GSD of air dried soils, which would facilitate the investigation of the disintegration of aggregates in WD and CD treatments.

**Fig 5:**
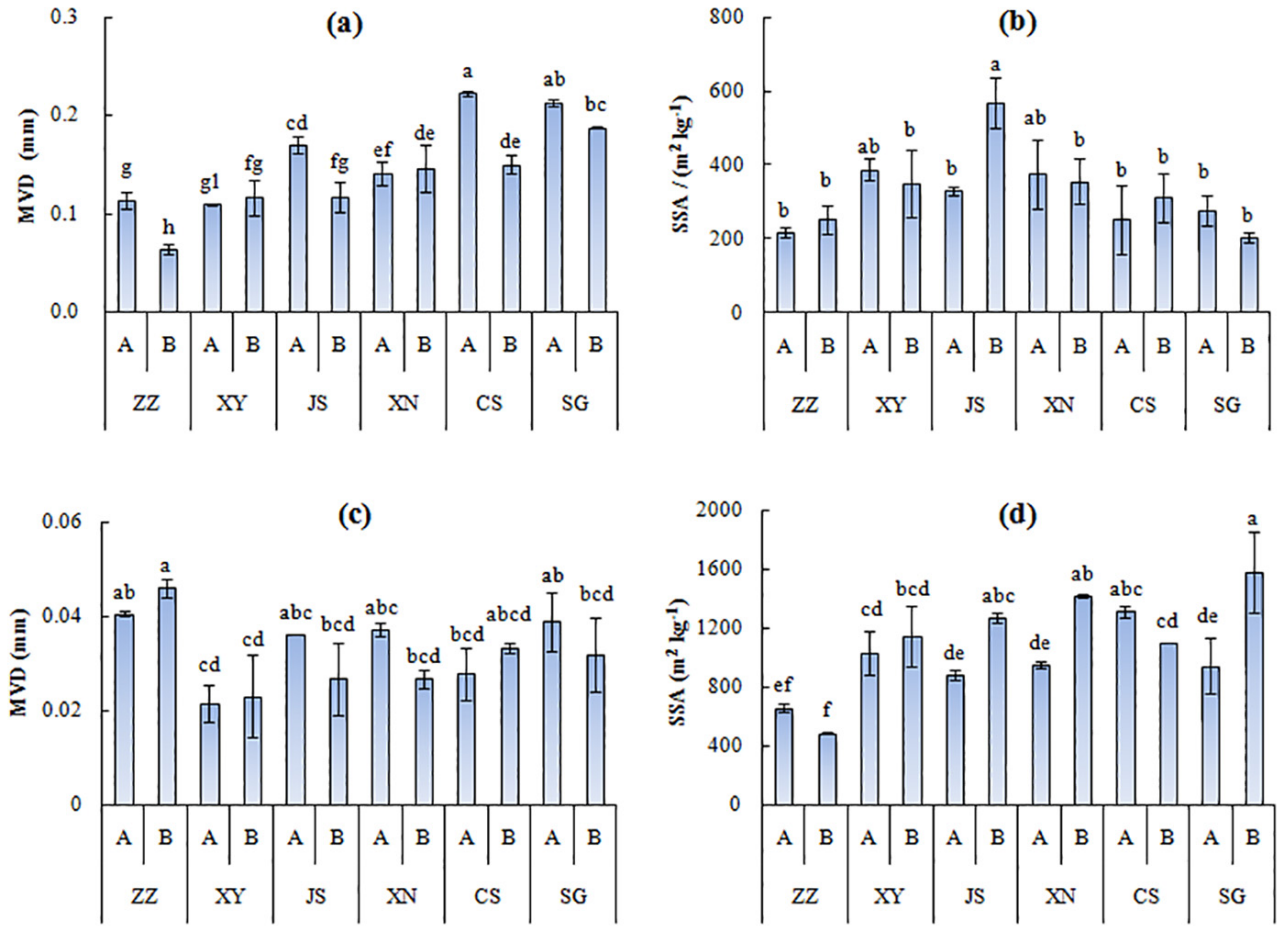
Mean volume diameter (MVD) and specific surface area (SSA) of different zonal soils: (a) MVD in water dispersion (WD) treatment;(b) SSA in WD treatment; (c)MVD in chemical dispersion (CD) treatment; (d) SSA in CD treatment.

Fig 5 depicts the variations of MVD and SSA in WD and CD treatments. The effects of treatment, soil type, horizons and their interaction on MVD and SSA were extremely significant (p<0.01) (Table 5). The MVD and SSA values in CD treatment were much smaller and larger than those in WD treatment, respectively. In the WD treatment, the MVD was overall smaller in B horizon than in A horizon (p<0.01) except for XY and XN, and the interaction of soil × horizon influenced the MVD (p<0.01) significantly. The values of MVD_WD_ generally increased with increasing soil weathering (Fig 5a). SSA_WD_ was significantly (p<0.05) affected by soil types, and this parameter was the largest for soil JS, but no significant differences were observed in the other soils, indicating a similar release rate of the fine grains in the disintegration process of air-dried aggregates in WD treatment (Fig 5b).

**Table 5:** ANOVA results for the effects of soil type, horizon, treatment and their interactions on the aggregate stability.

Index	Source	df	F	p
MVD	WD			
	Soil	5	58.7	<0.0001
	Horizon	1	37.2	<0.0001
	Soil×horizon	5	7.1	0.0033
	CD			
	Soil	5	8.0	0.0021
	Horizon	1	1.0	0.3336
	Soil×horizon	5	3.8	0.0296
	Total			
	Treatment	1	1834.6	<0.0001
	Soil	5	45.9	<0.0001
	Horizon	1	36.0	<0.0001
	Treatment×soil	5	50.7	<0.0001
	Soil×horizon	5	5.2	0.003
	Treatment×horizon	1	24.3	<0.0001
	Treatment×soil×horizon	5	6.9	0.0005
SSA	WD			
	Soil	5	4.0	0.0225
	Horizon	1	0.9	0.3482
	Soil×horizon	5	1.7	0.2068
	CD			
	Soil	5	16.0	0.0001
	Horizon	1	18.5	0.0013
	Soil×horizon	5	7.5	0.0037
	Total			
	Treatment	1	562.8	<0.0001
	Soil	5	14.3	<0.0001
	Horizon	1	15.4	0.0007
	Treatment×soil	5	10.9	<0.0001
	Soil×horizon	5	4.9	0.0032
	Treatment×horizon	1	8.7	0.0071
	Treatment×soil×horizon	5	6.1	0.001

Abbreviations: WD, water dispersion treatment; CD, chemical dispersion treatment; MVD, mean volume diameter; SSA, specific surface area.

In CD treatment, there was an extremely significant difference in MVD between soil types (p<0.01). MVDs of soil particles were the largest in ZZ soils, and the smallest in XY (Fig 5c). SSA_CD_ was significantly influenced by soil types, horizon and their interaction (p<0.01); SSA_CD_ was larger in A horizon than in B horizon. Contrary to MVD, SSA_CD_ in ZZ soils was the smallest and the other soils displayed no significant difference in SSA (Fig 5d).

**Fig 6:**
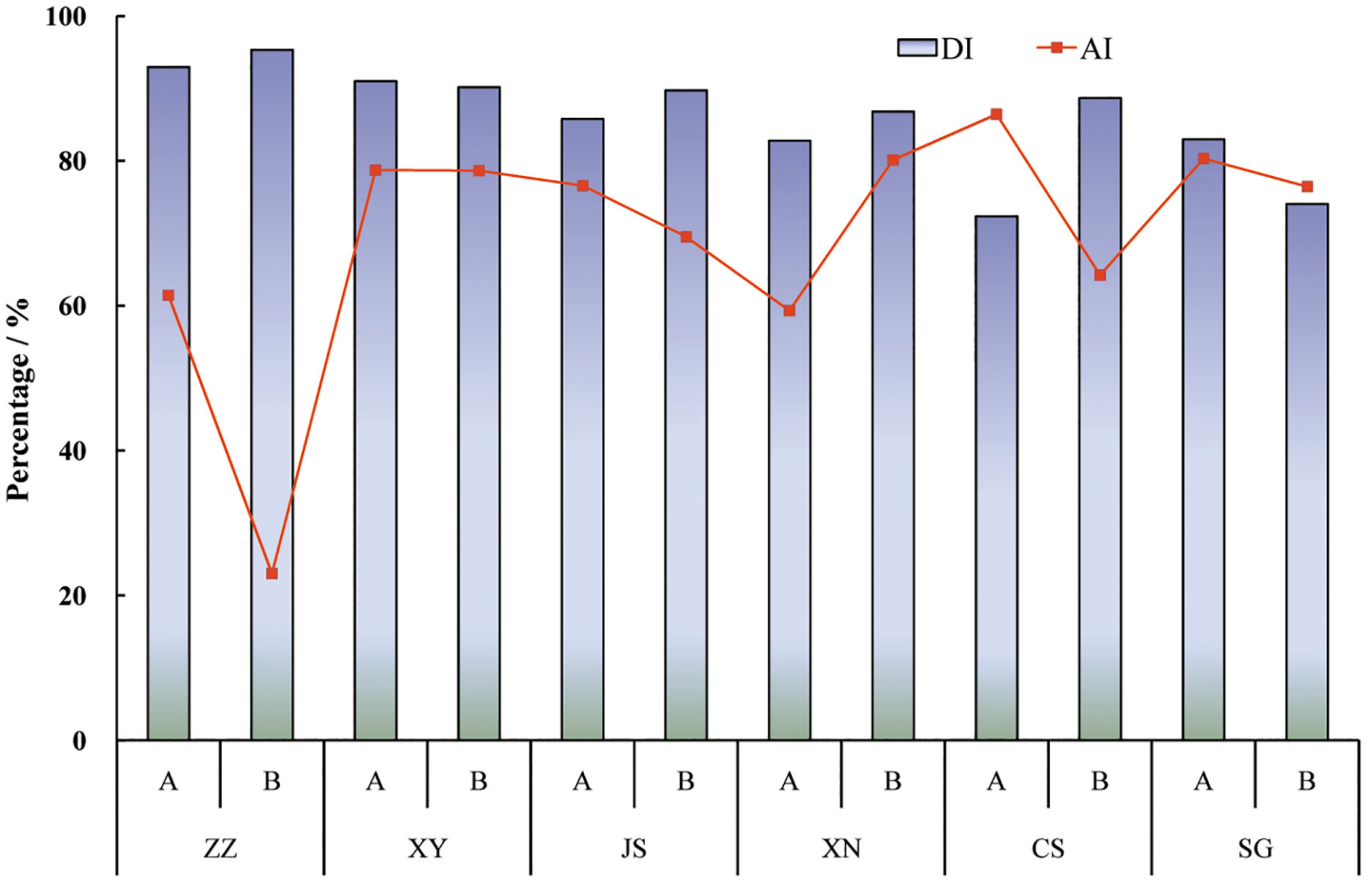
Detachability index (DI) and aggregation index (AI) of different zonal soils.

Detachability index (DI) decreased from 95% (ZZ-B) to 67% (CS-A and SG-B) (Fig 6). The larger value of DI indicates the lower soil water stability, which represents weaker resistance to water erosive force. Aggregation index (AI) ranged from 31 to 89% indicating remarkable differences in aggregation of soil particles, and the aggregation was found to be highest and lowest in CS-A and ZZ-B, respectively.

### Relationships between soil aggregation and physicochemical properties

Correlation analysis (Table 6) showed that MVD_WD_ had a significant positive correlation with SOM and different forms of sesquioxides (r≥0.57, p<0.05), but a negative one with pH (r = -0.60, p<0.05). Among these forms of sesquioxides, ln(Fe_o_-Fe_p_) had the most significant relationship with MVD_WD_ (p<0.001). SSA_WD_ was significantly (r = 0.65 and 0.69, p<0.05) related with CEC and BD, while the curvature coefficient (C_c_) was negatively correlated with CEC and BD (p<0.05). There was a significant correlation between the uniformity coefficient (C_u_) and different forms of sesquioxides except ln(Fe_p_) (p<0.05). DI was negatively related with ln(Fe_d_-Fe_o_), ln(Al_d_-Al_o_) and ln(Fe_o_-Fe_p_) (p<0.01), but positively with pH (p<0.05). AI was correlated positively with ln(Fe_o_-Fe_p_), ln(Al_o_-Al_p_), 2–50μm and SSA_CD_ (p<0.05), but negatively with MVD_CD_ and >50μm (p<0.05).

**Table 6:** Correlation coefficients between the indices of water stable aggregates and soil basic properties.

	MVD_WD_	SSA_WD_	C_u_	C_c_	DI	AI
pH	-0.60*	-	-	-	0.62*	-
SOM	0.61*	-	-	-	-	-
CEC	-	0.65*	-	-0.58*	-	-
BD	-	0.69*	-	-0.60*	-	-
ln(Fe_d_-Fe_o_)	0.77**	-	0.62*	-	-0.74**	0.61*
ln(Al_d_-Al_o_)	0.75**	-	0.76**	-	-0.72**	-
ln(Fe_o_-Fe_p_)	0.87***	-	0.73**	-	-0.73**	0.78**
ln(Al_o_-Al_p_)	0.57*	-	0.76**	-	-	0.81**
ln(Fe_p_)	0.60*	-	-	-	-	-
ln(Al_p_)	0.70*	-	0.73**	-	-0.65*	-
MVD_CD_	-	-	-	-	-	-0.60*
SSA_CD_	-	-	-	-	-0.65*	0.70*
<2 μm	-	-	-	-	-0.65*	0.71*
2–50μm	-	-	-	-	-0.58*	0.80**
>50μm	-	-	-	-	0.64*	-0.79**

*p<0.05;

**p<0.01;

***p<0.001.

BD, bulk density; CEC, cation exchange capacity; <2μm, 2~50μm and >50μm, the volume content (%) of particles (<2μm, 2~50μm and >50μm); Fe_d_ and Al_d_, free iron and aluminum oxides; Fe_o_ and Al_o_, amorphous iron and aluminum oxides; Fe_p_ and Al_p_, complex iron and aluminum oxides; MVD_CD_, and MVD_WD_, mean volume diameter of particles and water stable aggregates; C_u_ and C_c_ uniformity coefficient and curvature coefficient of water stable aggregates; DI, detachability index; AI, aggregation index; SSA_WD_ and SSA_CD_, surface specific area of water stable aggregates and particles.

**Fig 7:**
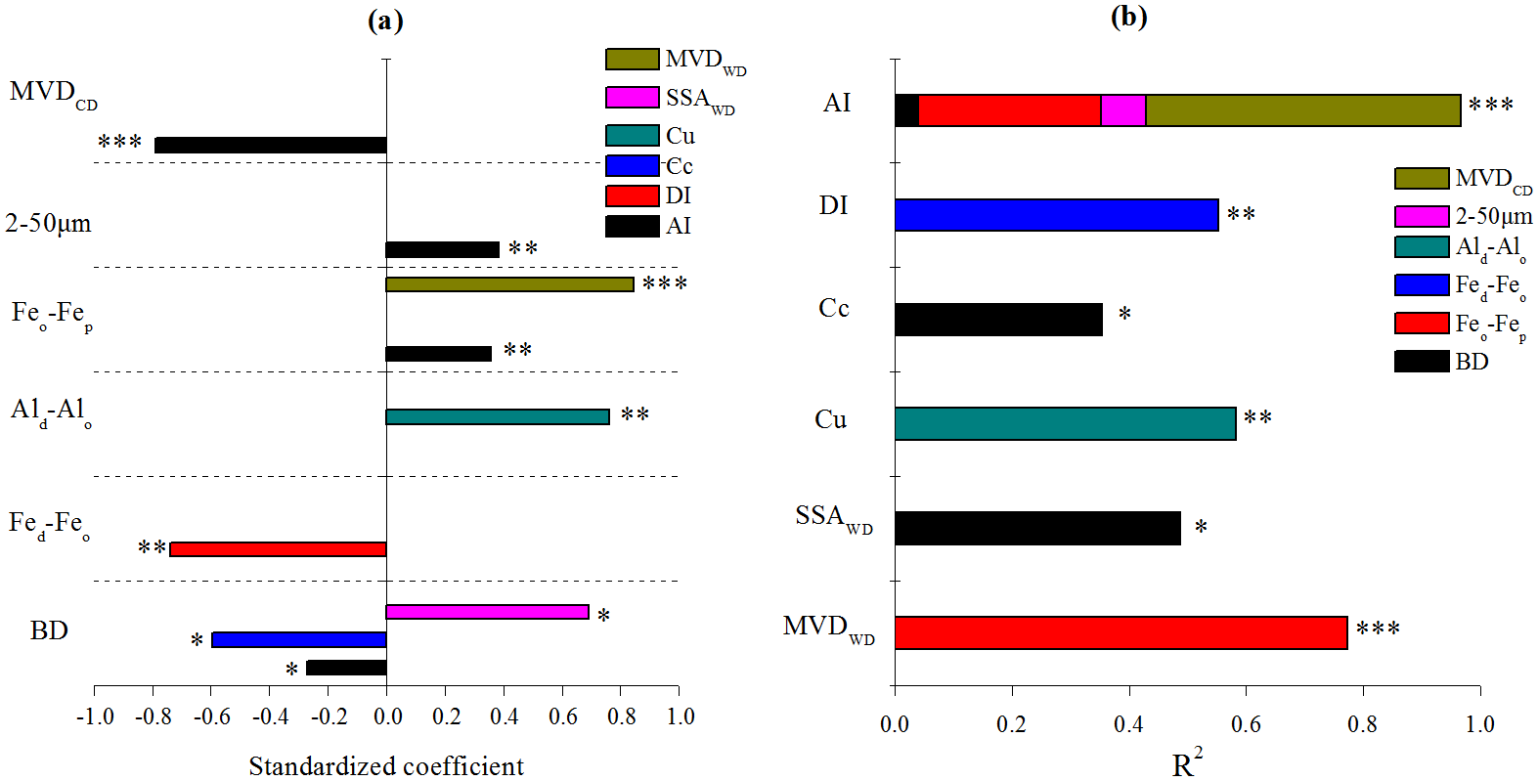
Standardized coefficients (a) and R^2^ (b) of entered variables in multiple stepwise regressions. (*, **, *** by the side of every bar represent the significant levels at p<0.05, 0.01 and 0.001 separately; BD, bulk density; 2~50μm, the volume of particles(2~50μm); Fe_d_ and Al_d_, free iron and aluminum oxides; Fe_o_ and Al_o_, amorphous iron and aluminum oxides; Fe_p_, complex iron oxides; MVD_WD_ and MVD_CD_, mean volume diameters of water stable aggregates and particles; C_u_, uniformity coefficient; C_c_, curvature coefficient; DI, detachability index; AI, aggregation index; SSA_WD_, surface specific area of water stable aggregates).

The results of multiple stepwise regressions are shown in Fig 7. Six independent variables (BD, 2~50 μm, Fe_d_-Fe_o_, Al_d_-Al_o_, Fe_o_-Fe_p_, MVD_CD_) (S1 Table) entered the models with low VIPs (<2) (S2 Table) at significant level of p<0.05. The ln(Fe_o_-Fe_p_) accounted for the majority of variations in MVD_WD_ (R^2^ = 77%) and BD explained 43% of the variance in SSA_WD_. The characteristics of water stable aggregates distribution (C_u_ and C_c_) were related to ln(Al_d_-Al_o_) and BD, respectively. Besides, DI was negatively related to (Fe_d_-Fe_o_) exponentially. AI was positively related with ln(Fe_o_-Fe_p_),but negatively with MVD_CD_, both of which explained 54 and 31% of its variance, respectively, followed by silt (2~50 μm) (8%) and BD (4%). It can be concluded that free oxide especially Fe_o_-Fe_p_ likely has the most significant influence on aggregate stability along the weathering gradient and that soil particle compositions (especially fine particles) and bulk density varied in their influence on the formation and stability of soil aggregates.

## Discussion

A high variability was observed in the iron and aluminum oxides, indicating that the tested soils varied in different weathering degrees in the subtropical-tropical regions. Aggregate water stability generally increased along the weathering gradient with the maximum value in CS-A. Meanwhile, the water stable aggregates size distribution gradually transformed to the large size (>500 μm). Under increasing disruptive forces, the peak of grain size distribution gradually changed towards small size to a different degree, indicating the stepwise fragmentation process, which is similar to the findings of Oades and Water [3]. When subjected to different disruptive forces, the soils in different weathering degrees generally displayed a similar aggregation process (Figs 3 and 4), further indicating that soil aggregation of these zonal soils along the weathering gradient conforms to aggregate hierarchy theory to some extent. Asano and Wagai proposed a conceptual model of aggregate hierarchy in Andisol at micron scales (<53 μm) [5]. More recent studies have shown the hierarchical structure in Oxisols analogous to the temperate soils [33, 34]. The main differences in aggregate breakdown among these soils lay in the amount of water stable aggregates. Miller and Schaetzl suggested that the clay-silt break be set at 6 μm in the grain measurement by laser diffraction [35]. Therefore, macroaggregates were assembled from sand- and silt- size grains (>20 μm) while water stable aggregates consisted of the particles of <40 μm.

In this paper, crystalline and amorphous iron, complex aluminum oxides and fine particles (<50μm) jointly contributed to aggregate water stability against slaking while pH and coarse particles (>50μm) played the inverse role. Imhoff et al. showed that clay and silt fractions (<50 μm) acted as cementing agent in water stable aggregate formation [36]. Lu et al. found that Fe_d_ and clay greatly influenced porosity in Ultisols [37]. The effect of crystalline oxides on soil porosity has been verified in another paper (Wu et al., unpublished). The aforementioned results suggest that crystalline oxides especially Fe_d_-Fe_o_, influenced aggregate formation and stability most likely through porosity. According to the degree of aggregate breakdown subjected to slaking, the binding effects of amorphous oxides and clay on the stability of macroaggregates (250~3000 μm) were relatively limited compared to crystalline oxides. Pronk et al. reported that the crystalline iron oxides are present as primary particles [38]. Peng et al. showed that organic matter played a primary role in aggregate stability (250–2000 μm) [11]. However, in the present study, we failed to observe the significant effect of SOM on aggregate stability, probably due to its small quantity in soils (below 23 g kg^-1^) [8].

When compared to the air dried macroaggregates, a higher similarity was observed in the formation of water stable aggregates, with both free sesquioxides and particle composition playing an important role in aggregation (Fig 5). Peng et al. showed that Fe/Al oxides seemed to be the major agents of <250 μm aggregates [11]. Huang et al. found that water stable macroaggregates (>250 μm) were correlated with the organic matter contents of eroded Ultisols [39]. In this study, water stable aggregates (SSA and MVD) and size distribution (C_u_, C_c_) were significantly related to pH, SOM, CEC, BD and free oxides, with ln(Fe_o_-Fe_p_), ln(Al_d_-Al_o_) and BD most closely linked to water stable aggregates along the weathering gradient. Duiker et al. reported that Fe_o_ and SOM were well correlated with aggregate stability [9].

It should be noteworthy that free oxides especially Fe_o_-Fe_p_ and Al_o_-Al_p_ and fine particles (<50 μm) were the dominant agents in aggregation along the weathering gradient. Likewise, the relative low content of organic matter (below 23 g kg^-1^) resulted in its less significant effects than sesquioxides and clay content on the formation of water stable aggregates [8]. The large surface area of Fe and Al oxides might facilitate reactions with clay particles through Coulombic forces [40]. Amorphous oxides exhibited the stronger cementing role in the aggregate formation than crystalline oxides. Previous studies showed that aggregate stability increased with an increase in clay content due to its cementing effect especially for the inactive clay mineralogy such as kaolinite [36, 41–43]. Overall, the ln(Fe_d_-Fe_o_), MVD_CD_ and ln(Fe_o_-Fe_p_) could be well used to evaluate and predict soil aggregation. As soil texture and sesquioxides are not easily managed at a small scale, organic matter could enhance soil aggregation, especially macroaggregate formation and stability. Further works are required to elucidate the mechanistic interaction between soil aggregation and these active agents.

## Conclusion

This study about the aggregation of zonal soils and its relationships with physicochemical properties from the temperate to topical regions indicates that, under increasing disruptive forces, soil aggregates showed a stepwise fragmentation and the peak of grain size distribution grain transformed to small size gradually. For zonal soils in different weathering degrees, the formation process of soils aggregates conformed to the aggregate hierarchy theory to some extent. Aggregate water stability generally increased with increasing soil weathering degree. Free oxides, organic matter, bulk density and particles composition exert their specific roles in different stages of soil aggregation. Crystalline (Fe_d_-Fe_o_) and amorphous iron oxides (Fe_o_-Fe_p_) in combination with fine particles (<50 μm) remarkably contributed to the formation and stability of air dried aggregates and water stable aggregates along the weathering gradient, respectively. However, the experiment evidence about the effects of sesquioxides and fine particles on soil aggregation remains to be explored in future studies.

## Supporting Information

S1 Fig X-ray diffractograms for oriented samples (Mg-ethylene glycol) of clay particles for these zonal soils. (K = kaolinite, Sm = smectite, Mi = hydromica, V = vermiculite, 1.4nm = 1.4nm intergrade mineral).
(TIF)

S1 Table Model coefficients of selected variables accounting for aggregate stability by multiple stepwise regression. (DOCX)

S2 Table Variance inflation factor (VIF) of selected variables accounting for aggregate stability by multiple stepwise regression. (DOCX)
