# Supplementary figures and images for: Variation of Soil Aggregation along the Weathering Gradient: Comparison of Grain Size Distribution under Different Disruptive Forces
Source: PLoS One. 2016 Aug 16;11(8):e0160960. doi: 10.1371/journal.pone.0160960 (PMC4986941; doi:10.1371/journal.pone.0160960)

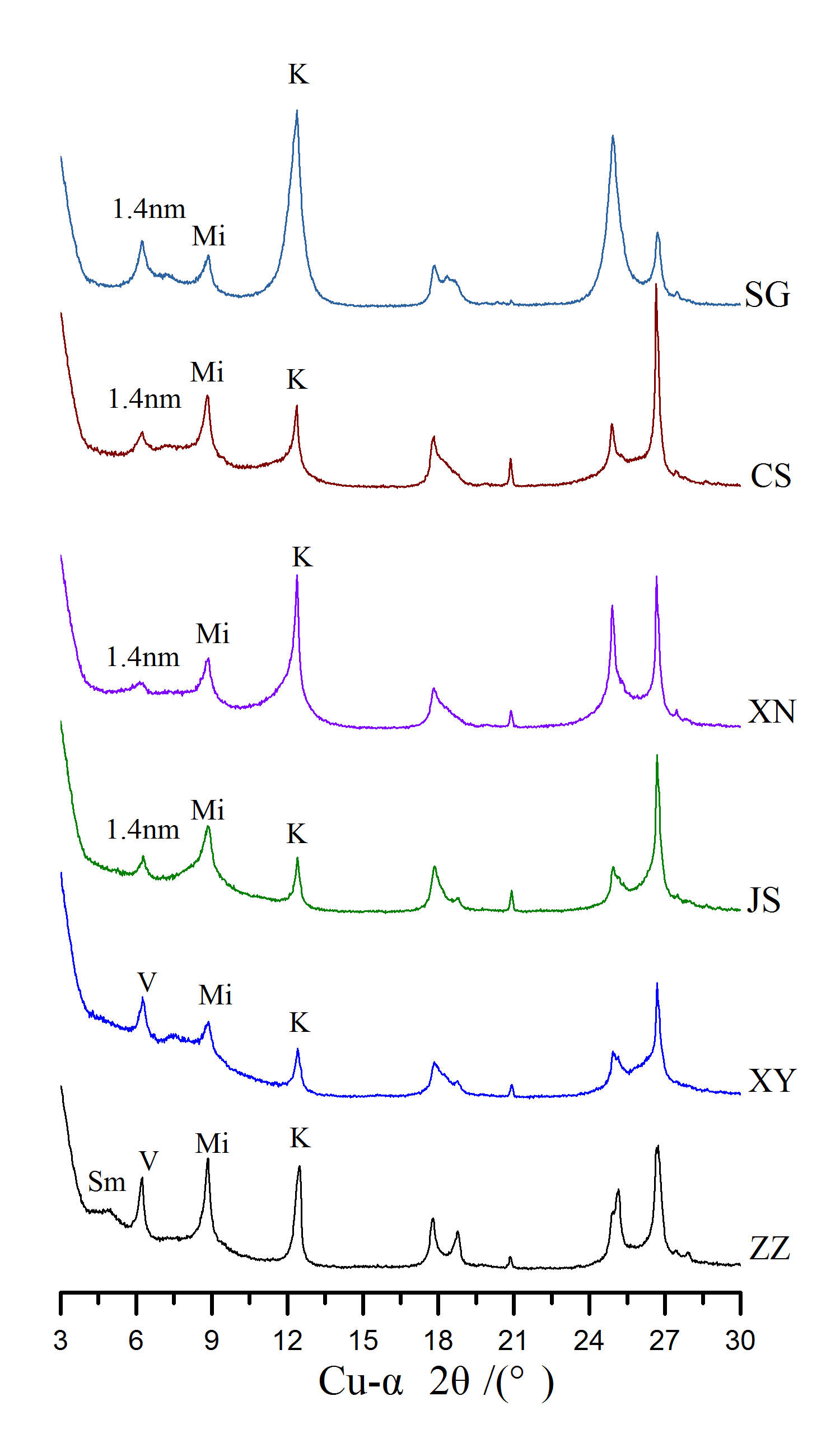

Supplement: S1 Fig — (K = kaolinite, Sm = smectite, Mi = hydromica, V = vermiculite, 1.4nm = 1.4nm intergrade mineral). (TIF) [file pone.0160960.s001.tif]
