# Supplementary material for: Variation of Soil Aggregation along the Weathering Gradient: Comparison of Grain Size Distribution under Different Disruptive Forces
Source: PLoS One. 2016 Aug 16;11(8):e0160960. doi: 10.1371/journal.pone.0160960 (PMC4986941; doi:10.1371/journal.pone.0160960)
Supplement: S1 Table — (DOCX) [file pone.0160960.s002.docx]

**S1 Table. Model coefficients of selected variables accounting for aggregate stability by multiple stepwise regressions**

| Variable | MVD_WD_ | SSA_WD_ | C_u_ | C_c_ | DI | AI |
| --- | --- | --- | --- | --- | --- | --- |
| Intercept | 0.07（0.01） | -421(242) | 12.5(1.1) | 3.0(0.8) | 104(5) | 19.5(20.1) |
| BD |  | 517(168) |  | -1.3(0.6) |  | -17.2(6.4) |
| ln(Fe_o_-Fe_p_) | 0.08(0.01) |  |  |  |  | 8.9(2.3) |
| ln(Fe_d_-Fe_o_) |  |  |  |  | -5.6(1.6) |  |
| ln(Al_d_-Al_o_) |  |  | 2.2(0.6) |  |  |  |
| ln(2~50μm) |  |  |  |  |  | 23.0(5.2) |
| MVD_CD_ |  |  |  |  |  | -584(62) |
| Adj-R^2^ | 0.75 | 0.44 | 0.54 | 0.29 | 0.51 | 0.95 |

Standardized error was in the parentheses; BD, bulk density; 2~50μm, the volume content (%) of particles (2~50μm); Fe_d_ and Al_d_, free iron and aluminum oxides; Fe_o_ and Al_o_, amorphous iron and aluminum oxides; Fe_p_, complex iron oxides; MVD_CD_, and MVD_WD_, mean volume diameters of particles, water stable aggregates; C_u_ , uniformity coefficient; C_c_, curvature coefficient ; DI, detachability index; AI, aggregation index; SSA_WD_, surface specific area of water stable aggregates.
