# Supplementary material for: Variation of Soil Aggregation along the Weathering Gradient: Comparison of Grain Size Distribution under Different Disruptive Forces
Source: PLoS One. 2016 Aug 16;11(8):e0160960. doi: 10.1371/journal.pone.0160960 (PMC4986941; doi:10.1371/journal.pone.0160960)
Supplement: S2 Table — (DOCX) [file pone.0160960.s003.docx]

**S2 Table. Variance inflation factor (VIF) of selected variables accounting for aggregate stability by multiple stepwise regressions.**

| Variable | MVD_WD_ | SSA_WD_ | C_u_ | C_c_ | DI | AI |
| --- | --- | --- | --- | --- | --- | --- |
| BD |  | 1.0 |  | 1.0 |  | 1.8 |
| ln(Fe_o_-Fe_p_) | 1.0 |  |  |  |  | 1.7 |
| ln(Fe_d_-Fe_o_) |  |  |  |  | 1.0 |  |
| ln(Al_d_-Al_o_) |  |  | 1.0 |  |  |  |
| ln(2~50μm) |  |  |  |  |  | 1.4 |
| MVD_CD_ |  |  |  |  |  | 1.3 |

BD, bulk density; 2~50μm, the volume content (%) of particles (2~50μm); Fe_d_ and Al_d_, free iron and aluminum oxides; Fe_o_ and Al_o_, amorphous iron and aluminum oxides; Fe_p_, complex iron oxides; MVD_CD_, and MVD_WD_, mean volume diameters of particles, water stable aggregates; C_u_ , uniformity coefficient; C_c_, curvature coefficient ; DI, detachability index; AI, aggregation index; SSA_WD_, surface specific area of water stable aggregates.
